# High use of antibiotics in elderly patients at discharge after hospitalization for acute abdominal pain

**DOI:** 10.1186/1757-7241-23-S1-A25

**Published:** 2015-07-16

**Authors:** Jakob S Jensen, Helle L Ipsen, Hanne Jørsboe

**Affiliations:** 1 Emergency Department, Nykøbing-Falster Hospital, Nykøbing-Falster, Denmark

## Background

8% of the annual discharges at our hospital of Nykøbing-Falster (NFS) are registered as acute abdominal pain. A former study in our department based on national data has demonstrated a high use of pain-killers, antibiotics, and antacids in these patients. We want to investigate if there is a difference in the drug consumption before and after hospitalization due to unspecific abdominal pain, in patients aged 18-60 compared to patients aged 80+.

## Methods

A retrospective audit study was performed in a group of younger patients (20-60 years) and a group of elderly (80+ years) with unresolved abdominal pain before and after admittance to NFS in 2012. Patients were included with a discharge code of acute abdominal pain without any explanation (ICD-10 code R10). We assessed the use of medication on the admission day and new drugs prescribed at discharge supplemented with duration of hospitalization, frequency of re-admittance within 30 days, 30 days mortality rate, and correct disease-coding. The following groups of drugs were studied; antibiotics, painkillers, and antacids including H2-antagonists. In 16 of 74 cases in the elderly and 3 of 74 cases in the young, we found a specific diagnosis and their data was excluded.

## Results

The study included 71 patients (20-60 years), mean age 37 years, and 58 patients (80-99 years) mean age 85 years. The 80+ year patients versus the younger had a longer length of stay, at 4.67 days (1-65) versus 2 days (1-12), readmission rate was higher at 28% versus 10.8% and mortality rate was 19% versus 0%. In general, the elderly were prescribed more medication at arrival and increased at discharge compared to the younger. The elderly had a significantly increase in new antibiotics from 7% at admission to 28% at discharge.

## Conclusion

We found that the elderly patients had a very high mortality rate. They were discharged without explanation for their abdominal pain, but had prescribed more new symptomatic medication as painkillers and antacids compared to the younger. As a new finding, we showed that the elderly in 28% of the cases were treated with antibiotics at discharge. Whether this is a bias to wrong disease coding or to symptomatic use of antibiotics with weak indications need further investigation.

